# Development of ambulatory care sensitive conditions (ACSC) in Thai context: hospitalisation rates for ACSC as indicator of access and quality in primary care

**DOI:** 10.1186/1471-2458-14-S1-O28

**Published:** 2014-01-29

**Authors:** Arnat Wannasri, Sirinard Nipaporn, Phatthanawilai Inmai, Thaworn Sakunphanit, Samrit Srithamrongsawat, Paibul Suriyawongpaisal

**Affiliations:** 1 Faculty of Medicine, Naresuan University, Phitsanulok 65000, Thailand; 2 Health Insurance System Research Office (HISRO), Nonthaburi 11000, Thailand; 3 Faculty of Medicine Ramathibodi Hospital, Mahidol University, Bangkok 10400, Thailand

## 

Healthcare infrastructures in Thailand have been developed for many decades to improve access to health care and equity of service utilisation across the country. Health care system is generally organised into three levels: primary care, secondary care and tertiary care. Primary care deals with curative care of common conditions, health promotion, disease prevention and rehabilitation. Given the scope of services and their proximity to households, primary care is more readily available to the community than higher levels of care. Recent publications emphasise the need to develop and specify the indicators to measure the performance of primary care. In many countries, hospitalisation rates for ambulatory care sensitive conditions (ACSC) are used as a proxy for analysing the access and quality of primary care; poor access and low quality of primary care contribute to higher hospitalisation rates with ACSC. The aim of this study was to develop an ACSC list in Thai context based on consensus among Thai health professionals due to the fact that this tool has not at present been established in Thailand.

The inpatient database of National Health Security Scheme, Social Security Scheme, and Civil Servant Medical Benefit Scheme in 2012 will be used to develop ACSC list as an indicator of access and quality of primary care. The ACSC criteria proposed by Solberg and Weissman will be applied to obtain a list of ACSC codes. The ACSC criteria are 1) hospitalisation rates of at least 1/10,000 populations; 2) clarity in definition and ICD-10 coding and; 3) potentially preventable or avoidable through primary care services. Fifteen health professionals who have experiences with primary care system more than 10 years will be invited to participate in both first and second rounds within the consensus process to judge the relationships between ACSC and primary care. In each round of the consensus process, the Kappa test will be performed to analyse the level of consensus in order to recruit the ACSC with Kappa score of 0.41 and higher.

